# Supplementary material for: Characterisation of between-cluster heterogeneity in malaria cluster randomised trials to inform future sample size calculations
Source: Nat Commun. 2025 Jul 18;16:6615. doi: 10.1038/s41467-025-61502-w (PMC12274344; doi:10.1038/s41467-025-61502-w)
Supplement: Supplementary file 1 — Supplementary Information [file 41467_2025_61502_MOESM1_ESM.pdf]

# Supplementary information

## Characterisation of between-cluster heterogeneity in malaria cluster randomised trials to inform future sample size calculations

---

### Table of Contents


Supplementary Table 1 .......... 2
Supplementary Table 2 .......... 3
Supplementary Table 3 .......... 4
Supplementary Table 4 .......... 5
Supplementary Table 5 .......... 6
Supplementary Figure 1 .......... 7
Supplementary Figure 2 .......... 8
Supplementary Figure 3 .......... 9
Supplementary Figure 4 .......... 10
Supplementary Table 6 .......... 11
Supplementary Table 7 .......... 12
Supplementary Figure 5 .......... 13
Supplementary Table 8 .......... 14
Supplementary Table 9 .......... 15
Supplementary Table 10 .......... 16

**Supplementary Table 1**: List of trials that provided data in this meta analysis.Data source details which trials provided data directly or were obtained from an online repository: ClinEpiDB.

| Trial # | First author | Year published | DOI | Trial registration # | Data source |
|---|---|---|---|---|---|
| 1 | Poespoprodjo JR | 2021 | 10.1016/S1473-3099(21)00358-3 | NCT02787070 | ClinEpiDB |
| 2 | Bath D | 2021 | 10.1016/S0140-6736(21)00251-8 | NCT02556242 | Provided by authors |
| 3 | Vilakati S | 2021 | 10.1136/bmjgh-2021-005021 | NCT02315690 | Provided by authors |
| 4 | Sternberg E | 2021 | 10.1016/S0140-6736(21)00250-6 | ISRCTN18145556 | Provided by authors |
| 5 | Chaccour C | 2021 | 10.1186/s12936-021-03611-7 | NCT02910934 | ClinEpiDB |
| 6 | Dabira ED | 2021 | 10.1016/S1473-3099(21)00557-0 | NCT03576313 | Provided by authors |
| 7 | Accrombessi M | 2021 | 10.1016/S0140-6736(22)02319-4 | NCT03931473 | Provided by authors |
| 8 | Mosha J | 2021 | 10.1016/S0140-6736(21)02499-5 | NCT03554616 | Provided by authors |
| 9 | Hsiang MS | 2020 | 10.1016/S0140-6736(20)30470-0 | NCT02610400 | Provided by authors |
| 10 | Staedke S | 2020 | 10.1016/S0140-6736(20)30214-2 | ISRCTN17516395 | ClinEpiDB |
| 11 | Syafruddin SG | 2020 | 10.4269/ajtmh.19-0554 | NCT02294188 | Provided by authors |
| 12 | Eisele TP | 2020 | 10.4269/ajtmh.19-0659 | NCT02329301 | Provided by authors |
| 13 | Arzika AM | 2019 | 10.1371/journal.pmed.1002835 | NCT02048007 | ClinEpiDB |
| 14 | Foy BD | 2019 | 10.1016/S0140-6736(18)32321-3 | NCT02509481 | Provided by authors |
| 15 | von Seidlein L | 2019 | 10.1371/journal.pmed.1002745 | NCT01872702 | Provided by authors |
| 16 | Protopopoff N | 2018 | 10.1016/S0140-6736(18)30427-6 | NCT02288637 | Provided by authors |
| 17 | Tiono AB | 2018 | 10.1016/S0140-6736(18)31711-2 | ISRCTN21853394 | Provided by authors |
| 18 | Kafy HT | 2017 | 10.1073/pnas.1713814114 | NCT01713517 | Provided by authors |
| 19 | Bradley J | 2016 | 10.1186/s12936-016-1433-0 | | Provided by authors |
| 20 | Bousema T | 2016 | 10.1371/journal.pmed.1001993 | NCT01575613 | Provided by authors |
| 21 | West PA | 2014 | 10.1371/journal.pmed.1001630 | NCT01697852 | Provided by authors |
| 22 | Corbel V | 2012 | 10.1016/S1473-3099(12)70081-6 | ISRCTN07404145 | Provided by authors |
| 23 | Rowland M | 2001 | 10.1016/S0140-6736(00)04955-2 | | Provided by authors |
| 24 | Rowland M | 2000 | 10.1046/j.1365-3156.2000.00581.x | | Provided by authors |

**Supplementary Table 2**: Overall characteristics of the trials that provided cluster-level data in this meta- analysis.

| Trial # | First author | Country | Intervention type | Study arms (n) | Prevalence data | Incidence data |
|---|---|---|---|---|---|---|
| 1 | Poespoprodjo 2021 | Indonesia | Drug | 2 | | Included |
| 2 | Bath 2021 | South Africa | Vector | 2 | | Included |
| 3 | Vilakati 2021 | Eswatini | Drug | 2 | | Included |
| 4 | Sternberg 2021 | Côte d'Ivoire | Vector | 2 | Included | Included |
| 5 | Chaccour 2021 | Mozambique | Vector | 2 | Included | Included |
| 6 | Dabira 2021 | The Gambia | Vector | 2 | Included | Included |
| 7 | Accrombessi 2021 | Benin | Vector | 3 | Included | |
| 8 | Mosha 2021 | Tanzania | Vector | 4 | Included | Included |
| 9 | Hsiang 2020 | Namibia | Drug & vector | 4 | Included | Included |
| 10 | Staedke 2020 | Uganda | Vector | 2 | Included | |
| 11 | Syafruddin 2020 | Indonesia | Vector | 2 | | Included |
| 12 | Eisele 2020 | Zambia | Drug | 3 | Included | |
| 13 | Arzika 2019 | Niger | Drug | 2 | Included | |
| 14 | Foy 2019 | Burkina Faso | Drug | 2 | | Included |
| 15 | von Seidlein 2019 | Laos, Cambodia, Vietnam & Myanmar | Drug | 2 | Included | Included |
| 16 | Protopopoff 2018 | Tanzania | Vector | 4 | Included | |
| 17 | Tiono 2018 | Burkina Faso | Vector | 2 | Included | |
| 18 | Kafy 2017 | Sudan | Vector | 2 | Included | Included |
| 19 | Bradley 2016 | Equatorial Guinea | Vector | 2 | Included | |
| 20 | Bousema 2016 | Kenya | Drug & vector | 2 | Included | |
| 21 | west 2014 | Tanzania | Vector | 2 | Included | |
| 22 | Corbel 2012 | Benin | Vector | 4 | Included | Included |
| 23 | Rowland 2001 | Pakistan | Vector | 2 | Included | Included |
| 24 | Rowland 2000 | Pakistan | Vector | 3 | Included | |

**Supplementary Table 3**: Characteristics of malaria CRTs included in this meta-analysis compared to the characteristics of the malaria CRTs included in the original systematic review.

| Trial characteristics | Meta-analysis N=24 | | Systematic review N=71 | |
|---|---|---|---|---|
| | % | n | % | n |
| Trial location | | | | |
| Africa | 79.2% | 19 | 70.4% | 50 |
| Asia | 20.8% | 5 | 26.8% | 19 |
| America | 0.0% | 0 | 2.8% | 2 |
| Trial publication date | | | | |
| 1990-1999 | 0.0% | 0 | 2.8% | 2 |
| 2000-2009 | 8.3% | 2 | 18.3% | 13 |
| 2010-2019 | 41.7% | 10 | 52.1% | 37 |
| post 2020 | 50.0% | 12 | 26.8% | 19 |
| Intervention type | | | | |
| Vector control | 66.7% | 16 | 54.9% | 39 |
| Chemoprevention | 25.0% | 6 | 36.6% | 26 |
| Combination | 8.3% | 2 | 7.0% | 5 |
| Other | 0.0% | 0 | 1.4% | 1 |
| Primary outcome | | | | |
| Incidence | 58.3% | 14 | 54.9% | 39 |
| Prevalence | 33.3% | 8 | 45.1% | 32 |
| Study arms (#) | | | | |
| 2 | 70.8% | 17 | 77.5% | 55 |
| 3 | 12.5% | 3 | 12.7% | 9 |
| 4 | 16.7% | 4 | 9.9% | 7 |

**Supplementary Table 4**: Characteristics of the cluster-level prevalence data for malaria CRTs provided in this meta-analysis. Trial endemicity categorised according to the overall control-arm prevalence reported throughout the trial.

| Trial # | Trial | Surveys (n) | Clusters (n) | Cluster size (mean) | Diagnostic used | Age range tested (years) | Intervention coverage data | Powered for prevalence[a] | Overall control arm prevalence[b] | Endemicity |
|---|---|---|---|---|---|---|---|---|---|---|
| 4 | Sternberg 2021 | 2 | 40 | 47.5 | RDT | 0_10 | Included | Yes | 0.66 | High |
| 5 | Chaccour 2021 | 2 | 89 | 8.7 | RDT | All_ages | Included | | 0.42 | High |
| 6 | Dabira 2021 | 3 | 32 | 123.3 | PCR | All_ages | | Yes | 0.13 | Medium |
| 7 | Accrombessi 2021 | 1 | 61 | 74 | RDT | All_ages | Included | Yes | 0.43 | High |
| 8 | Mosha 2021 | 6 | 86 | 54.5 | RDT | 0.5_14 | Included | Yes | 0.37 | Medium |
| 9 | Hsiang 2020 | 1 | 56 | 80.6 | PCR | All_ages | | Yes | 0.01 | Low |
| 10 | Staedke 2020 | 4 | 104 | 77.3 | microscopy | 2_10 | | Yes | 0.15 | Medium |
| 12 | Eisele 2020 | 3 | 60 | 41.5 | RDT | 0_6 | | Yes | 0.15 | Medium |
| 13 | Arzika 2019 | 3 | 30 | 37.2 | microscopy | 0_5 | | Yes | 0.08 | Low |
| 15 | von Seidlein 2019 | 1 | 16 | 421.6 | PCR | All_ages | | Yes | 0.06 | Low |
| 16 | Protopopoff 2018 | 7 | 48 | 80.1 | RDT | 0.5_14 | Included | Yes | 0.52 | High |
| 17 | Tiono 2018 | 4 | 40 | 78.2 | microscopy | 0.5_5 | Included | | 0.53 | High |
| 18 | Kafy 2017 | 3 | 26 | 125.7 | RDT | 0.5_10 | | | 0.06 | Low |
| 19 | Bradley 2016 | 2 | 24 | 93.8 | RDT | 2_14 | Included | Yes | 0.19 | Medium |
| 20 | Bousema 2016 | 3 | 10 | 138 | PCR | All_ages | | Yes | 0.15 | Medium |
| 21 | West 2014 | 5 | 51 | 84.8 | RDT | 0.5_14 | Included | Yes | 0.17 | Medium |
| 22 | Corbel 2012 | 2 | 28 | 347.6 | microscopy | 0_6 | | | 0.26 | Medium |
| 23 | Rowland 2001 | 3 | 6 | 1064 | microscopy | All_ages | | | 0.01 | Low |
| 24 | Rowland 2000 | 2 | 9 | 239.4 | microscopy | 5_15 | | | 0.01 | Low |

a: powered according to sample size calculation which accounted for clustering

b: mean cluster-level Incidence per person per year (pp py) in the control arm throughout the trial

**Supplementary Table 5**: Characteristics of the cluster-level incidence data for malaria CRTs provided in this meta-analysis Trial endemicity categorised according to the overall control-arm incidence reported throughout the trial.

| Trial # | Trial | Method of case detection | Study years (n) | Clusters (n) | Cluster person-years (mean) | Diagnostic used | Age range tested (years) | Powered for incidence[a] | Overall control arm incidence[b] | Endemicity |
|---|---|---|---|---|---|---|---|---|---|---|
| 1 | Poespoprodjo 2021 | Active | 1 | 21 | 7.6 | microscopy | All | Yes | 0.84 | Medium |
| 2 | Bath 2021 | Passive | 2 | 62 | 6345 | RDT & micros. | All | Yes | 0.001 | Low |
| 3 | Vilakati 2021 | Passive | 2 | 76 | 1423.7 | RDT & micros. | All | Yes | 0.003 | Low |
| 4 | Sternberg 2021 | Active | 2 | 40 | 40.7 | RDT | 0.5-10 | Yes | 2.236 | High |
| 5 | Chaccour 2021 | Active | 2 | 83 | 12.1 | RDT | 0-5 | Yes | 5.346 | Medium |
| | | Passive | 2 | 187 | 743 | RDT | All | | 0.464 | Medium |
| 6 | Dabira 2021 | Passive | 1 | 32 | 154 | PCR | All | | 0.161 | Low |
| 8 | Mosha 2021 | Active | 2 | 86 | 34.5 | RDT | 0.5-10 | Yes | 0.447 | Medium |
| 9 | Hsiang 2020 | Passive | 1 | 56 | 241.5 | RDT & micros. | All | Yes | 0.041 | Low |
| 11 | Syafruddin 2020 | Active | 3 | 24 | 33.8 | PCR | All | Yes | 0.346 | Medium |
| 14 | Foy 2019 | Active | 1 | 8 | 25.5 | RDT | 0_5 | Yes | 6.924 | High |
| 15 | von Seidlein 2019 | Active | 1 | 16 | 327.5 | PCR | All | | 0.209 | Low |
| 18 | Kafy 2017 | Active | 3 | 26 | 221.6 | RDT | 0.5-10 | Yes | 0.043 | Low |
| 22 | Corbel 2012 | Active | 1 | 28 | 6 | microscopy | 0-6 | Yes | 0.879 | High |
| 23 | Rowland 2001 | Passive | 3 | 6 | 7794.6 | microscopy | All | | 0.008 | Low |

a: powered according to sample size calculation which accounted for clustering

b: mean cluster-level Incidence per person per year (pp py) in the control arm throughout the trial

**Supplementary Figure 1**: Distribution of the cluster-level prevalence (a), ACD incidence (b) and PCD incidence (c) outcomes stratified by trial. ACD: Active case detection incidence. PCD: Passive case detection incidence.

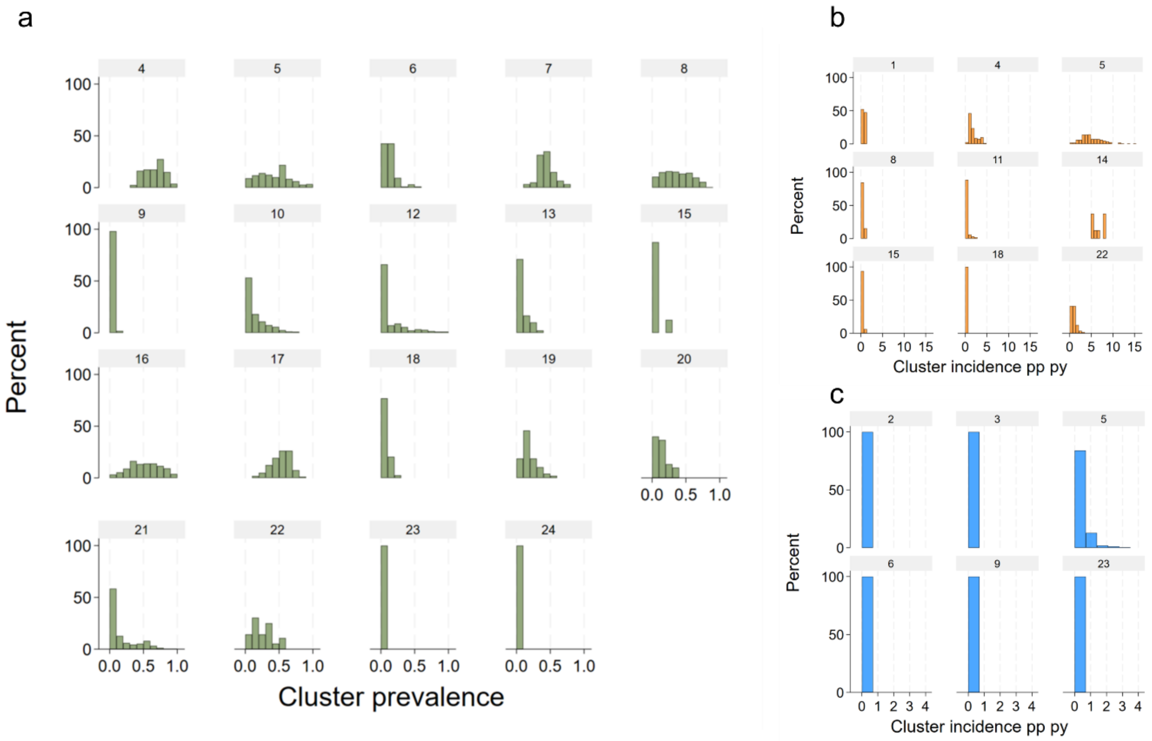

**Supplementary Figure 2**: The agreement between the methods of moments and random effects model approaches for estimating prevalence k (coefficient of variation) (green) and incidence k (red) for each survey-arm and study year-arm, respectively. Mixed: mixed effects linear regression approach. xtpoisson: mixed effects poisson regression approach.

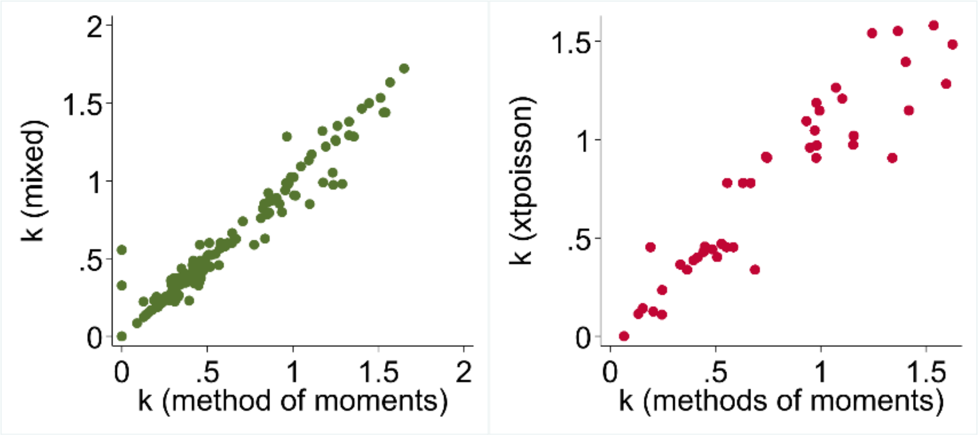

**Supplementary Figure 3**: Arm-differential patterns in prevalence k and ICC over time stratified by overall survey prevalence among malaria CRTs. A positive k/ICC difference indicates between cluster heterogeneity is higher in the intervention arm. A negative K/ICC difference indicates between cluster heterogeneity is higher in the control arms. Horizontal dashed line equals no differences in k/ICC between arms. Vertical dashed lines indicate the time the intervention was implemented.

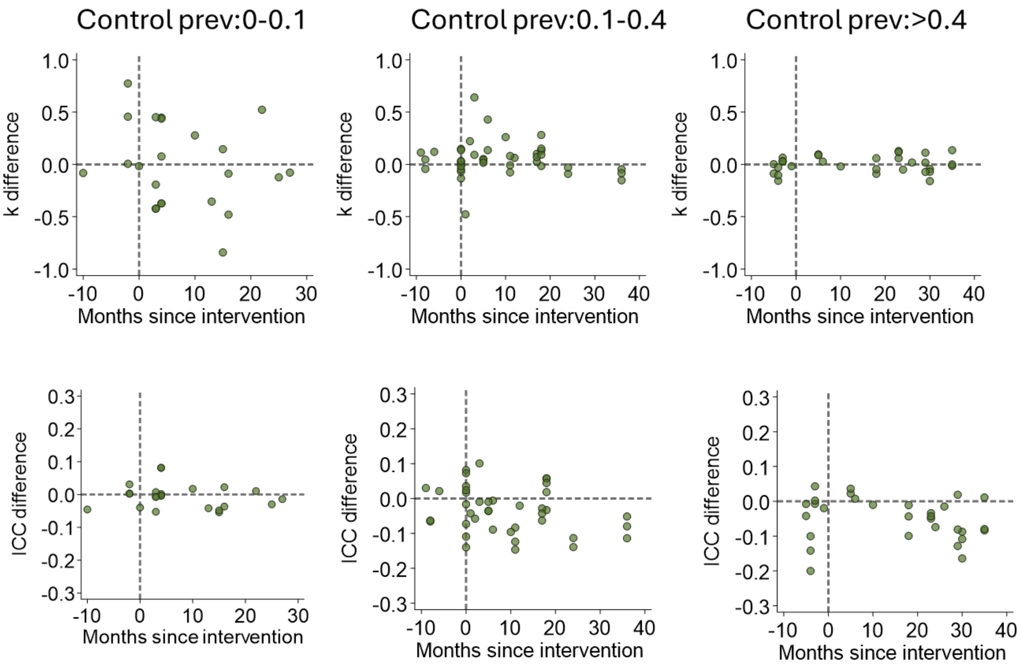

**Supplementary Figure 4**: Temporal patterns in between-cluster heterogeneity in malaria CRTs. **A**: Prevalence k estimates (dots) in the intervention arms of repeated surveys among trials. **B**: Incidence k estimates (dots) in the control arm of each study year among trials. **C**: Incidence k estimates (dots) in the intervention arm of each study year among trials. **D**: Prevalence ICC estimates (dots) in the intervention arms of repeated surveys among trials. Dashed lines refer to individual trials. ACD: active case detection incidence. PCD: Passive case detection incidence. Error bars represent 95%CIs around k estimates.

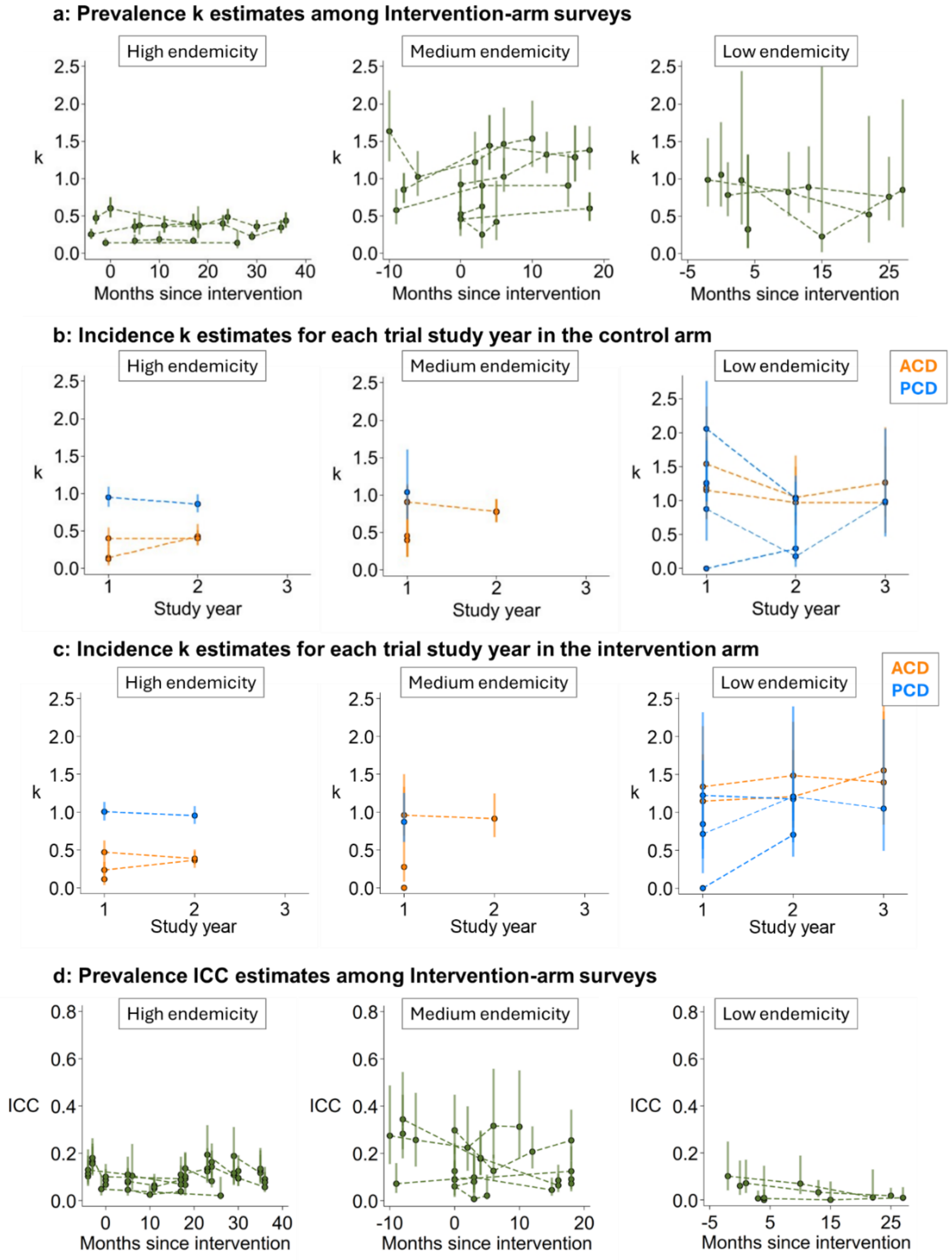

**Supplementary Table 6**: Observed prevalence and incidence coefficient of variation (k) estimates in the control arms of malaria CRTs that measured both outcome measures. Bold highlight signifies highest k estimate for given trial time periods.

| Trial | Prevalence | | | Active incidence | | | Passive incidence | | |
|---|---|---|---|---|---|---|---|---|---|
| # | Survey year | k | [95%CI] | Cohort year | k | [95%CI] | Cohort year | k | [95%CI] |
| 4 | 2016 | 0.15 | [0.11-0.23] | | | | | | |
| | | | | 2017-18 | 0.24 | [0.16-0.35] | | | |
| | 2018 | 0.12 | [0.07-0.22] | 2018-19 | **0.37** | **[0.26-0.51]** | | | |
| 5 | | | | | | | 2016-17 | 1.01 | [0.89-1.14] |
| | 2017 | 0.33 | [0.21-0.53] | 2017-18 | 0.47 | [0.35-0.63] | 2017-18 | **0.95** | **[0.84-1.08]** |
| | 2018 | 0.26 | [0.13-0.52] | 2018-19 | **0.39** | **[0.31-0.48]** | | | |
| 6 | 2017 | 0.47 | [0.3-0.74] | | | | | | |
| | 2018 | 0.35 | [0.21-0.57] | | | | | | |
| | 2019 | 0.79 | [0.55-1.15] | 2019-20 | **0.87** | **[0.61-1.25]** | | | |
| 8 | 2018 | 0.43 | [0.31-0.6] | | | | | | |
| | 2019 | 0.66 | [0.48-0.93] | 2019-20 | **0.96** | **[0.69-1.33]** | | | |
| | 2020 | 0.38 | [0.27-0.53] | 2020-21 | **0.91** | **[0.67-1.25]** | | | |
| | 2021 | 0.53 | [0.38-0.73] | | | | | | |
| | 2021 | 0.45 | [0.32-0.63] | | | | | | |
| | 2022 | 0.52 | [0.37-0.73] | | | | | | |
| 9 | 2017 | 0.37 | [0.12-1.14] | | | | 2017 | **0.85** | **[0.52-1.38]** |
| 15[a] | 2014 | **1.26** | **[0.77-2.08]** | 2014-15 | 1.15 | [0.75-1.76] | | | |
| 18 | 2012 | 1.29 | [0.86-1.96] | 2012-13 | **1.36** | **[0.82-2.27]** | | | |
| | 2013 | **1.35** | **[0.89-2.06]** | 2013-14 | 1.21 | [0.80-1.82] | | | |
| | 2014 | 0.80 | [0.5-1.26] | 2014-15 | **1.55** | **[1.00-2.41]** | | | |
| 22 | 2008 | 0.39 | [0.22-0.69] | 2008-09 | **0.45** | **[0.16-1.21]** | | | |
| | 2009 | 0.44 | [0.26-0.75] | | | | | | |
| 23 | 1995 | 0.63 | [0.2-1.95] | | | | 1995 | **0.71** | **[0.39-1.68]** |
| | 1996 | 0.97 | [0.43-2.22] | | | | 1996 | **1.21** | **[0.61-2.4]** |
| | 1997 | 0.99 | [0.43-2.28] | | | | 1997 | **1.05** | **[0.49-2.23]** |

*a: k estimated across four study site countries*

**Supplementary Table 7**: Risk factors associated with elevated prevalence k values (k>0.5) among all trial cross-sectional survey arms. Odds ratios estimated using logistic regression models that accounted for random effects at the trial level. OR: odds ratio. aOR: adjusted odds ratio. P-values: 2-sided p-values

| Risk factor | | Elevated k (>0.5) | | | Univariate[a] | | | Multivariate[a] | | |
|---|---|---|---|---|---|---|---|---|---|---|
| | | % | N | n | OR | [95%CI] | p-value | aOR | [95%CI] | p-value |
| **Survey arm prevalence** | | | | | | | | | | |
| | <10% | 76.3 | 38 | 29 | (ref) | | | (ref) | | |
| | 10-40% | 58.1 | 62 | 36 | 0.346 | [ 0.034 - 3.58 ] | 0.373 | 0.284 | [ 0.026 - 3.05 ] | 0.299 |
| | ≥40% | 2.0 | 50 | 1 | 0.008 | [ <0.001 - 0.23 ] | 0.005 | 0.015 | [ <0.001 - 0.563 ] | 0.023 |
| **Individuals tested per cluster** | | | | | | | | | | |
| | <60 | 44.4 | 45 | 20 | (ref) | | | | | |
| | ≥60 | 43.8 | 105 | 46 | 0.348 | [ 0.017 - 7.02 ] | 0.491 | | | |
| **Clusters per arm** | | | | | | | | | | |
| | <15 | 38.2 | 76 | 29 | (ref) | | | | | |
| | ≥15 | 50.0 | 74 | 37 | 0.474 | [ 0.002 - 99.9 ] | 0.784 | | | |
| **Study Arm** | | | | | | | | | | |
| | Control | 48.2 | 56 | 27 | (ref) | | | | | |
| | Intervention | 41.5 | 94 | 39 | 1.789 | [ 0.499 - 6.42 ] | 0.372 | | | |
| **Months since intervention** | | | | | | | | | | |
| | Pre-intervention | 50.0 | 44 | 22 | (ref) | | | | | |
| | 0-15 | 53.7 | 54 | 29 | 1.127 | [ 0.193 - 6.58 ] | 0.895 | | | |
| | >15 | 28.8 | 52 | 15 | 0.638 | [ 0.156 - 2.61 ] | 0.532 | | | |
| **Season** | | | | | | | | | | |
| | Non-malaria | 28.3 | 46 | 13 | (ref) | | | (ref) | | |
| | Malaria | 51.0 | 104 | 53 | 18.156 | [ 2.345 - 14.6 ] | 0.005 | 7.33 | [ 1.032 - 55.2] | 0.047 |
| **Diagnostic** | | | | | | | | | | |
| | PCR | 33.3 | 18 | 6 | (ref) | | | | | |
| | Microscopy/RDT | 45.5 | 132 | 60 | 13.25 | [ 0.027 - 6.54 ] | 0.414 | | | |
| **Total** | | | 150 | | | | | | | |

*a: logisitic regression model with random effects at the trial level adjusted for season and overall survey-arm prevalence.*

**Supplementary Figure 5**: Survey-level k values in the control arm and effect size (prevalence ratios) estimations by months since intervention introduction among eight trials that conducted surveys in both malaria and non-malaria seasons. Trials 12, 39 and 47 experienced larger effect sizes (lower prevalence ratios) in the malaria seasons when k was higher. Dots represent k estimates. Crosses represent prevalence ratios. Error bars represent 95%CIs.

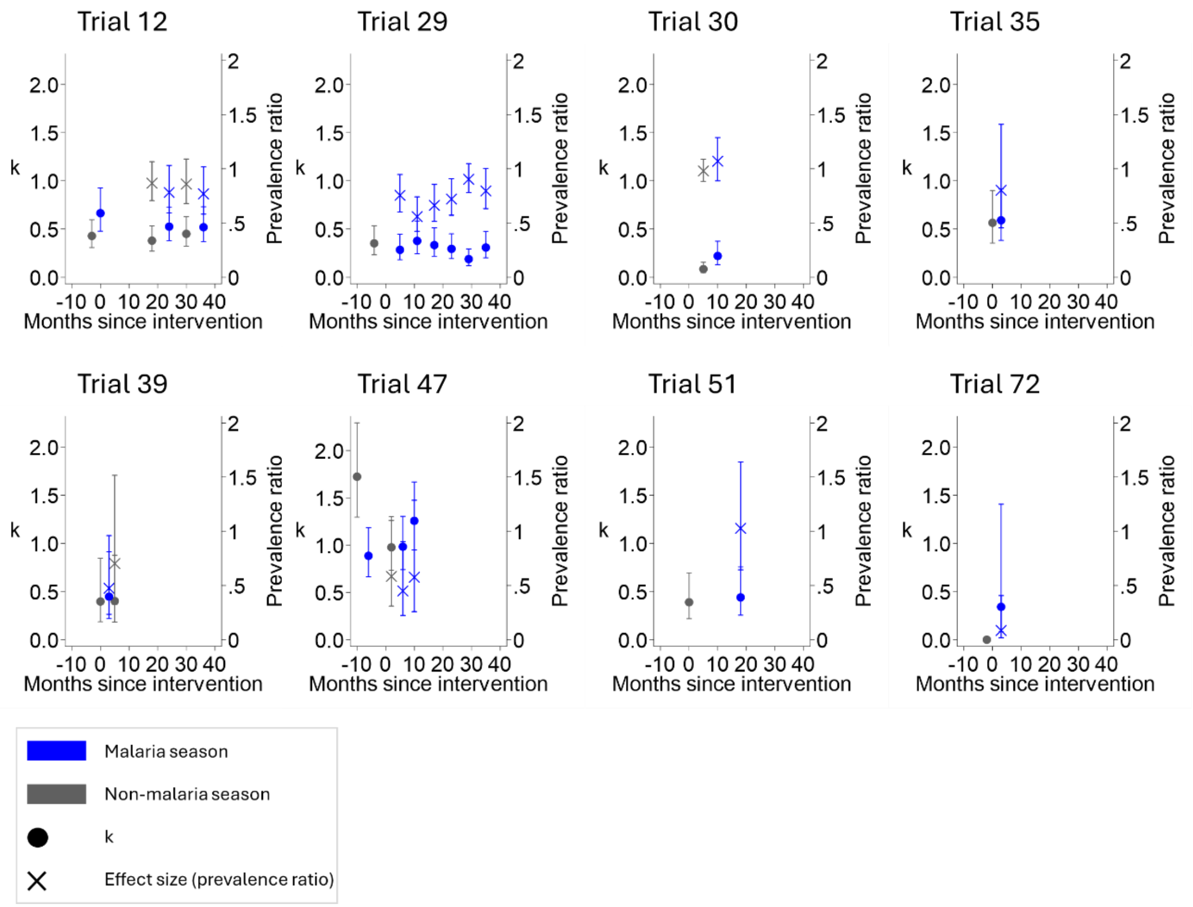

**Supplementary Table 8**: Dummy tables used to obtain prevalence data from malaria CRTs

| Trial ID | Survey number | Cluster | Arm allocation | Individuals surveyed (N) | Malaria positive (n) | Intervention users (n) |
|---|---|---|---|---|---|---|
| ## | ## | ## | ## | ## | ## | ## |
| ## | ## | ## | ## | ## | ## | ## |
| ## | ## | ## | ## | ## | ## | ## |
| ## | ## | ## | ## | ## | ## | ## |
| ## | ## | ## | ## | ## | ## | ## |
| ## | ## | ## | ## | ## | ## | ## |
| ## | ## | ## | ## | ## | ## | ## |
| ## | ## | ## | ## | ## | ## | ## |
| ## | ## | ## | ## | ## | ## | ## |

**Supplementary Table 9**: Dummy tables used to obtain active case detection (ACD) incidence data from malaria CRTs

| Trial ID | Survey number | Cluster | Arm allocation | Person-years at risk (N) | New malaria cases (n) |
|---|---|---|---|---|---|
| ## | ## | ## | ## | ## | ## |
| ## | ## | ## | ## | ## | ## |
| ## | ## | ## | ## | ## | ## |
| ## | ## | ## | ## | ## | ## |
| ## | ## | ## | ## | ## | ## |
| ## | ## | ## | ## | ## | ## |
| ## | ## | ## | ## | ## | ## |
| ## | ## | ## | ## | ## | ## |
| ## | ## | ## | ## | ## | ## |

**Supplementary Table 10**: Dummy tables used to obtain passive case detection (PCD) incidence data from malaria CRTs

| Trial ID | Survey number | Cluster | Arm allocation | Population at risk (N) | New malaria cases (n) |
|---|---|---|---|---|---|
| ## | ## | ## | ## | ## | ## |
| ## | ## | ## | ## | ## | ## |
| ## | ## | ## | ## | ## | ## |
| ## | ## | ## | ## | ## | ## |
| ## | ## | ## | ## | ## | ## |
| ## | ## | ## | ## | ## | ## |
| ## | ## | ## | ## | ## | ## |
| ## | ## | ## | ## | ## | ## |
| ## | ## | ## | ## | ## | ## |
