# Supplementary material for: Characterisation of between-cluster heterogeneity in malaria cluster randomised trials to inform future sample size calculations
Source: Nat Commun. 2025 Jul 18;16:6615. doi: 10.1038/s41467-025-61502-w (PMC12274344; doi:10.1038/s41467-025-61502-w)
Supplement: Supplementary file 3 — Description of Additional Supplementary Files [file 41467_2025_61502_MOESM3_ESM.pdf]

Description of Additional Supplementary Files

File Name: Supplementary Data 1
Description: STATA code used to estimate the coefficient of variation and intra-cluster correlation coefficient of prevalence outcomes.

File Name: Supplementary Data 2
Description: STATA code used to estimate coefficient of variation of incidence outcomes

File Name: Supplementary Data 3
Description: Fictious cluster-level prevalence data

File Name: Supplementary Data 4
Description: Fictious cluster-level incidence data
