# Supplementary material for: Characterisation of between-cluster heterogeneity in malaria cluster randomised trials to inform future sample size calculations
Source: Nat Commun. 2025 Jul 18;16:6615. doi: 10.1038/s41467-025-61502-w (PMC12274344; doi:10.1038/s41467-025-61502-w)
Supplement: Supplementary file 4 — Supplementary Data 1 [file 41467_2025_61502_MOESM4_ESM.docx]

////////////////////////////////////////////////////////////////////////////////

///////Prevalence coefficient of variation (k) estimation: methods of moments////

////////////////////////////////////////////////////////////////////////////////

//Code description//

*Code illustrates methods-of-moments approach to estimate the coefficient of

*variation of cluster-level prevalence data at the survey-arm level.

//Required data//

*Code requires cluster-level prevalence data. For illustrative purposes use

*supplementary data 3

//Trial data variable description//

*Trial_no = unique trial identifier

*Survey_no = unique survey identifier for each trial

*Cluster_no = unique cluster identifier for each trial

*Arm_no = trial arm (0: control, 1: Intervention)

*prev = the cluster-level prevalence

*n_pos = number of positive cases per cluster

*N_tested = total number of cases tested per cluster

//Code//

**Visualise cluster-level prevalence data at survey arm level:

hist prev, by(Survey_no Arm_no)

**cluster average prevalence by survey arm:

bysort Survey_no Arm_no: egen cluster_mean_prev=mean(prev)

**Overall average prevalence by arm:

bysort Survey_no Arm_no: egen overall_n_pos=sum(n_pos)

bysort Survey_no Arm_no: egen overall_n_tested=sum(N_tested)

gen Overall_mean_prev=overall_n_pos/overall_n_tested

drop overall_n_pos overall_n_tested

**Harmonic mean of cluster size:

bysort Survey_no Arm_no: egen count_tested=count(N_tested)

bysort Survey_no Arm_no: gen one_over_n=1/N_tested

bysort Survey_no Arm_no: egen sum_one_over_n=sum(one_over_n)

bysort Survey_no Arm_no: gen harmean_cluster_N= count_tested/sum_one_over_n

drop count_tested one_over_n sum_one_over_n

**Sum square differences:

gen prev_mean_difference=prev-cluster_mean_prev

gen difference2=prev_mean_difference*prev_mean_difference

bysort Trial_no Survey_no Arm_no: egen sum_of_squares=sum(difference2)

drop difference2 prev_mean_difference

**Cluster n-1:

bysort Survey_no Arm_no: egen c_no=count(Cluster_no)

gen c_no_minus_1=c_no-1

drop c_no

**Estimate k:

gen s2=sum_of_squares/c_no_minus_1

gen variance=s2-((Overall_mean_prev*(1-Overall_mean_prev))/harmean_cluster_N)

gen SD=sqrt(variance)

gen k=SD/Overall_mean_prev

drop SD variance s2 c_no_minus_1 sum_of_squares harmean_cluster_N ///

Overall_mean_prev cluster_mean_prev

**END***************************************************************************

////////////////////////////////////////////////////////////////////////////////

///////Incidence coeffcient of variation (k) estimation: methods of moments/////

////////////////////////////////////////////////////////////////////////////////

//Code description//

*Code illustrates methods-of-moments approach to estimate the coefficient of

*variation of cluster-level incidence data at the study year-arm level.

//Required data//

*Code requires cluster-level incidence data. For illustrative purposes, use

*supplementary data 4

//Trial data variable description//

*Trial_no = unique trial identifier

*Study_year = unique study year identifier for each trial

*Cluster_no = unique cluster identifier for each trial

*Arm_no = trial arm (0: control, 1: Intervention)

*inci = the cluster-level incidence per person per year

*n_pos = number of new positive cases per cluster

*N_personyears = total person years at risk per cluster

//Code//

**Visualise cluster-level incidence data at study year-arm level:

hist inci, by(Study_year Arm_no)

**cluster average inci:

bysort Study_year Arm_no: egen cluster_mean_inci=mean(inci)

***Overall inci:

bysort Study_year Arm_no: egen overall_n_pos=sum(n_pos)

bysort Study_year Arm_no: egen overall_personyears=sum(N_personyears)

gen Overall_inci=overall_n_pos/overall_personyears

drop overall_n_pos overall_personyears

**Harmonic mean of cluster personyears:

bysort Study_year Arm_no: egen count_tested=count(N_personyears)

bysort Study_year Arm_no: gen one_over_n=1/N_personyears

bysort Study_year Arm_no: egen sum_one_over_n=sum(one_over_n)

bysort Study_year Arm_no: gen harmean_cluster_py= count_tested/sum_one_over_n

drop count_tested one_over_n sum_one_over_n

**Sum square differences:

gen inci_mean_difference=inci-cluster_mean_inci

gen difference2=inci_mean_difference*inci_mean_difference

bysort Study_year Arm_no: egen sum_of_squares=sum(difference2)

drop difference2 inci_mean_difference

**Cluster n-1:

bysort Study_year Arm_no: egen c_no=count(Cluster_no)

gen c_no_minus_1=c_no-1

drop c_no

**Estimate k:

gen s2=sum_of_squares/c_no_minus_1

gen BC_var=s2-((Overall_inci)/harmean_cluster_py)

gen BC_SD=sqrt(BC_var)

gen k=BC_SD/Overall_inci

drop BC_SD BC_var s2 c_no_minus_1 sum_of_squares harmean_cluster_py ///

Overall_inci cluster_mean_inci

**END***************************************************************************

////////////////////////////////////////////////////////////////////////////////

///////Prevalence coeffcient of variation (k) estimation: Regression////////////

////////////////////////////////////////////////////////////////////////////////

//Code description//

*Code illustrates regression approach to estimate the coefficent of

*variation, and corresponding 95%CI, of cluster-level prevalence data at the ///

*survey-arm level. It starts by expanding the cluster-level prevalence data to /

> //

*the individual level.

//Required data//

*Code requires cluster-level prevalence data. For illustrative purposes use

*supplementary data 3

//Trial data variable description//

*Trial_no = unique trial identifier

*Survey_no = unique survey identifier for each trial

*Cluster_no = unique cluster identifier for each trial

*Arm_no = trial arm (0: control, 1: Intervention)

*prev = the cluster-level prevalence

*n_pos = number of positive cases per cluster

*N_tested = total number of cases tested per cluster

//code//

**expand cluster-level prevalence data to individual level:

*gen unque id for expanding:

gen survey_arm_cluster_no=string(Survey_no,"%02.0f") + string(Arm_no,"%02.0f") +

> string(Cluster_no,"%03.0f")

*expand to individual level data: generate binary var: case (1/0)

expandcl N_tested, gen(newcl) cluster(survey_arm_cluster_no)

bysort survey_arm_cluster_no: gen obs = _n

bysort survey_arm_cluster_no: replace obs =. if obs>n_pos

drop n_pos

gen case=0

replace case=1 if !missing(obs)

drop obs newcl

**create unique id to run model over each survey-arm:

gen surveyarm=string(Survey_no,"%02.0f") + string(Arm_no,"%02.0f")

destring surveyarm, generate(surveyarm_no)

drop surveyarm

**estimate k, and upper and lower 95%CI, for each survey arm:

gen variance =.

gen low_variance =.

gen up_variance =.

gen k=.

gen up_k=.

gen low_k=.

levelsof surveyarm_no, local(tsa)

foreach q of local tsa {

mixed case if surveyarm_no==`q' || Cluster_no:

matrix b = e(b)

matrix list b

replace variance = exp(_b[lns1_1_1:_cons])^2 if surveyarm_no==`q'

replace low_variance = exp(_b[lns1_1_1:_cons] - 1.96*_se[lns1_1_1:_cons]

> )^2 if surveyarm_no==`q'

replace up_variance = exp(_b[lns1_1_1:_cons] + 1.96*_se[lns1_1_1:_cons])

> ^2 if surveyarm_no==`q'

replace k=sqrt(exp(_b[lns1_1_1:_cons])^2)/(_b[case:_cons]) if surveyarm_

> no==`q'

replace up_k=sqrt(exp(_b[lns1_1_1:_cons] + 1.96*_se[lns1_1_1:_cons])^2)/

> (_b[case:_cons]) if surveyarm_no==`q'

replace low_k=sqrt(exp(_b[lns1_1_1:_cons] - 1.96*_se[lns1_1_1:_cons])^2)

> /(_b[case:_cons]) if surveyarm_no==`q'

}

**collapse data back to cluster level:

collapse ///

(mean) Trial_no Arm_no prev ///

(sum) case ///

(mean) N_tested k up_k low_k, ///

by(Cluster_no Survey_no)

sort Trial_no Survey_no Arm_no Cluster_no

**END***************************************************************************

////////////////////////////////////////////////////////////////////////////////

///////Incidence coeffcient of variation (k) estimation: Regression/////////////

////////////////////////////////////////////////////////////////////////////////

//Code description//

*Code illustrates poisson regression approach to estimate the coefficent of

*variation of cluster-level incidence data at the study year-arm level.

//Required data//

*Code requires cluster-level incidence data. For illustrative purposes, use

*supplementary data 4

//Trial data variable description//

*Trial_no = unique trial identifier

*Study_year = unique study year identifier for each trial

*Cluster_no = unique cluster identifier for each trial

*Arm_no = trial arm (0: control, 1: Intervention)

*inci = the cluster-level incidence per person per year

*n_pos = number of new positive cases per cluster

*N_personyears = total person years at risk per cluster

**create unique id to run model over each survey-arm:

gen yeararm= string(Study_year,"%02.0f") + string(Arm_no,"%02.0f")

destring yeararm, generate(yeararm_no)

***estiamte k & uncertainy from sqrt alpha for each trial, year and arm:

gen k=.

gen up_k=.

gen low_k=.

levelsof yeararm_no, local(sa)

foreach q of local sa {

xtpoisson n_pos if yeararm_no==`q', exposure(N_personyears) irr i(Cluste

> r_no)

matrix b = r(table)

matrix list b

replace k=sqrt(b[1,3]) if yeararm_no==`q'

replace up_k=sqrt(b[6,3]) if yeararm_no==`q'

replace low_k=sqrt(b[5,3]) if yeararm_no==`q'

}

sort Study_year Arm_no Cluster_no

**END***************************************************************************
