# Supplementary material for: Characterisation of between-cluster heterogeneity in malaria cluster randomised trials to inform future sample size calculations
Source: Nat Commun. 2025 Jul 18;16:6615. doi: 10.1038/s41467-025-61502-w (PMC12274344; doi:10.1038/s41467-025-61502-w)
Supplement: Supplementary file 5 — Supplementary Data 2 [file 41467_2025_61502_MOESM5_ESM.docx]

////////////////////////////////////////////////////////////////////////////////

///Prevalence Intracluster correlation coefficent (ICC) estimation: Regression//

////////////////////////////////////////////////////////////////////////////////

//Code description//

*Code illustrates regression approach to estimate the ICC (and 95%CI) using

*cluster-level prevalence data at the survey-arm level.

//Required data//

*Code requires cluster-level prevalence data. For illustrative purposes use

*supplementary data 3

//Trial data variable description//

*Trial_no = unique trial identifier

*Survey_no = unique survey identifier for each trial

*Cluster_no = unique cluster identifier for each trial

*Arm_no = trial arm (0: control, 1: Intervention)

*prev = the cluster-level prevalence

*n_pos = number of positive cases per cluster

*N_tested = total number of cases tested per cluster

//code//

**expand cluster-level prevalence data to individual level:

*gen unique id for expanding:

gen survey_arm_cluster_no=string(Survey_no,"%02.0f") + string(Arm_no,"%02.0f") + string(Cluster_no,"%03.0f")

*expand to individual level data: generate binary var: case (1/0)

expandcl N_tested, gen(newcl) cluster(survey_arm_cluster_no)

bysort survey_arm_cluster_no: gen obs = _n

bysort survey_arm_cluster_no: replace obs =. if obs>n_pos

drop n_pos

gen case=0

replace case=1 if !missing(obs)

drop obs newcl

**create unique id to run model over each survey-arm:

gen surveyarm=string(Survey_no,"%02.0f") + string(Arm_no,"%02.0f")

destring surveyarm, generate(surveyarm_no)

drop surveyarm

**estimate ICC, and upper and lower 95%CI, for each survey arm:

gen ICC=.

gen up_ICC=.

gen low_ICC=.

levelsof surveyarm_no, local(tsa)

foreach q of local tsa {

mixed case if surveyarm_no==`q' || Cluster_no:

estat icc

return list

scalar ICC_val = r(icc2)

replace ICC=ICC_val if surveyarm_no==`q'

matrix ci = r(ci2)

scalar low_ICC_val = ci[1,1]

replace low_ICC=low_ICC_val if surveyarm_no==`q'

scalar up_ICC_val = ci[1,2]

replace up_ICC=up_ICC_val if surveyarm_no==`q'

}

**collapse individual-level data back to cluster level:

collapse ///

(mean) Trial_no Arm_no prev ///

(sum) case ///

(mean) N_tested ICC up_ICC low_ICC, ///

by(Cluster_no Survey_no)

sort Trial_no Survey_no Arm_no Cluster_no

**END***************************************************************************
